# *Staphylococcus aureus* Induces Eosinophil Cell Death Mediated by α-hemolysin

**DOI:** 10.1371/journal.pone.0031506

**Published:** 2012-02-15

**Authors:** Lynne R. Prince, Kirstie J. Graham, John Connolly, Sadia Anwar, Robert Ridley, Ian Sabroe, Simon J. Foster, Moira K. B. Whyte

**Affiliations:** 1 Department of Infection and Immunity, University of Sheffield, Sheffield, United Kingdom; 2 Krebs Institute, Department of Molecular Biology and Biotechnology, University of Sheffield, Sheffield, United Kingdom; University of Liverpool, United Kingdom

## Abstract

*Staphylococcus aureus*, a major human pathogen, exacerbates allergic disorders, including atopic dermatitis, nasal polyps and asthma, which are characterized by tissue eosinophilia. Eosinophils, via their destructive granule contents, can cause significant tissue damage, resulting in inflammation and further recruitment of inflammatory cells. We hypothesised that the relationship between *S. aureus* and eosinophils may contribute to disease pathology. We found that supernatants from *S. aureus* (SH1000 strain) cultures cause rapid and profound eosinophil necrosis, resulting in dramatic cell loss within 2 hours. This is in marked contrast to neutrophil granulocytes where no significant cell death was observed (at equivalent dilutions). Supernatants prepared from a strain deficient in the accessory gene regulator (*agr*) that produces reduced levels of many important virulence factors, including the abundantly produced α-hemolysin (Hla), failed to induce eosinophil death. The role of Hla in mediating eosinophil death was investigated using both an Hla deficient SH1000-modified strain, which did not induce eosinophil death, and purified Hla, which induced concentration-dependent eosinophil death via both apoptosis and necrosis. We conclude that *S. aureus* Hla induces aberrant eosinophil cell death *in vitro* and that this may increase tissue injury in allergic disease.

## Introduction

Eosinophils and their products are major components of allergic inflammation in tissues, in particular in atopic dermatitis, rhinitis and asthma, where their abundance in the tissues and circulation correlates with disease severity [1], [2], [3], [4]. As granule-containing leukocytes, eosinophils can damage tissue cells via the release of toxic mediators including eosinophil cationic protein (ECP), major basic protein, eosinophil-derived neurotoxin and eosinophil peroxidase (EPO). These mediators can directly damage the airway epithelium, induce mast cell histamine release and increase airway responsiveness via contraction of smooth muscle [5], [6]. Mediator release can occur following degranulation, as part of the innate immune response, but also via cell rupture. In the absence of an activation stimulus eosinophils undergo constitutive apoptosis, in the same way as the shorter-lived neutrophil granulocytes, and this is followed by clearance via tissue macrophages [7]. This anti-inflammatory process limits the destruction mediated by intracellular proteases and promotes the resolution of inflammation. The process of immune cell apoptosis can be dysregulated by pathogens, which are well known to disrupt the death pathways of both monocyte-macrophages and neutrophils as a survival strategy [8], [9], [10]. Similarly eosinophils, as key anti-parasitic cells, are targeted by parasites, which induce premature eosinophil apoptosis as a mechanism of immune evasion [11], [12], [13]. Although eosinophils are not usually considered to play a significant role in immune defences against bacteria, some studies have shown they possess anti-bacterial capabilities, mediated by their granule contents [14], [15] and the more recently discovered release of mitochondrial DNA [16].

The important human pathogen, *S. aureus*, exacerbates the pathology of allergic diseases that are typically characterised by tissue eosinophilia, in particular atopic dermatitis [17], [18], nasal polyps [19], [20] and asthma [21]. *S. aureus* produces a number of virulence factors pertinent to its survival in the host, notably cytolysins which include the hemolysins ( α, β, γ and δ) and Panton-Valentine leukotoxin (PVL). It is unclear, however, how the relationship between *S. aureus* and eosinophils may contribute to disease and the effects of *S. aureus* upon eosinophil viability had not been examined. In these studies we show *S. aureus* mediates rapid eosinophil cell death and that the cytolysin Hla is a major contributory factor in eosinophil death.

## Materials and Methods

### Bacterial information and culture

The *Staphylococcus aureus* wild type (WT) SH1000 strain and its corresponding *agr* derivative were used [22]. The Hla deficient SH1000 *hla* strain was created from DU1090 [23] via transduction [24] using phage φ11 and selection for erythromycin (5 µg/ml) and lincomycin (25 µg/ml) resistance. *S. aureus* was grown on LB plates (supplemented with tetracycline 5 µg/ml for *agr* and erythromycin 5 µg/ml and lincomycin 25 µg/ml for *hla*) from which single colonies were picked and inoculated into 5 ml LB broth for an overnight shaking culture (18 hr). For complementation of SH1000 *hla* to yield the strain *hla* ^+hla^, the *hla* gene plus an 812-bp sequence upstream of the open reading frame was amplified using primers F-ApaI and R-BamHI (see table 1). The fragment was cloned as an ApaI/BamHI fragment into similarly cut pGL485 [25], and the resulting construct (pJC001) and pGL485 (control) were transformed into *S. aureus* RN4220 by selection on chloramphenicol [26]. Phage transduction [24]) using φ11 was used to transfer pJC001 and pGL485 into SH1000 and SH1000 *hla*. Successful complementation was confirmed by colony PCR, using plasmid specific primers covering the insert region (ALB23 and ALB24, see table 1). Bacterial yield was determined to be comparable for all cultures by serial dilution and plating onto LB plates. Culture supernatants from liquid cultures were produced by removal of bacteria by repeated microcentrifugation at 6000 RPM and filtration (0.45 µm). Hla (60% protein content by Lowry activity ≥10,000 units/mg protein) was obtained from Sigma-Aldrich (Dorset, UK).

**Table 1:** Oligonucleotides used for complementation.

Oligonucleotide	Sequence 5′-3′
F-ApaI	ATAATAGGGCCCACTCTGTCATTTTTAATCCCCTTTC
R-BamHI	ATAATAGGATCCAAACCATTTGTTAACCTCCTTGAAC
ALB23	TGTGAGGGGATAACAATTCCCC
ALB24	TTCCTTTCGGGCTTTGTTAGCAG

### Preparation and culture of eosinophils

Granulocytes were isolated from fresh whole blood from healthy human volunteers by dextran sedimentation and plasma-percoll centrifugation [27]. All subjects gave informed consent and ethical approval was given by the Sheffield Research Ethics Committee. Purity was assessed by counting >500 cells on duplicate slides and granulocytes were typically 5–15% eosinophils. In selected experiments, eosinophils were further purified from granulocyte populations by negative magnetic selection using a human eosinophil enrichment kit and RoboSep technology (StemCell Technologies, Vancouver, BC, Canada) yielding populations of >95% eosinophils. Cells were cultured at a density of 2.5×10^6^/ml in RPMI 1640 supplemented with 10% FCS and 1% penicillin/streptomycin, in 96 well polypropylene plates (Becton Dickinson) at 37°C/5% CO_2_.

### Assessment of cell death

Light microscopy: Eosinophils display characteristic morphological nuclear changes during apoptosis that can be visualised by light microscopy [7]. Cytocentrifuge slides (cytospins, Shandon) were prepared from cell cultures at the time points indicated, fixed and stained with modified Wright-Giemsa stain (Diff-Quik). Apoptosis was assessed by counting >100 eosinophils per slide of duplicate pairs.

**Figure 1:**
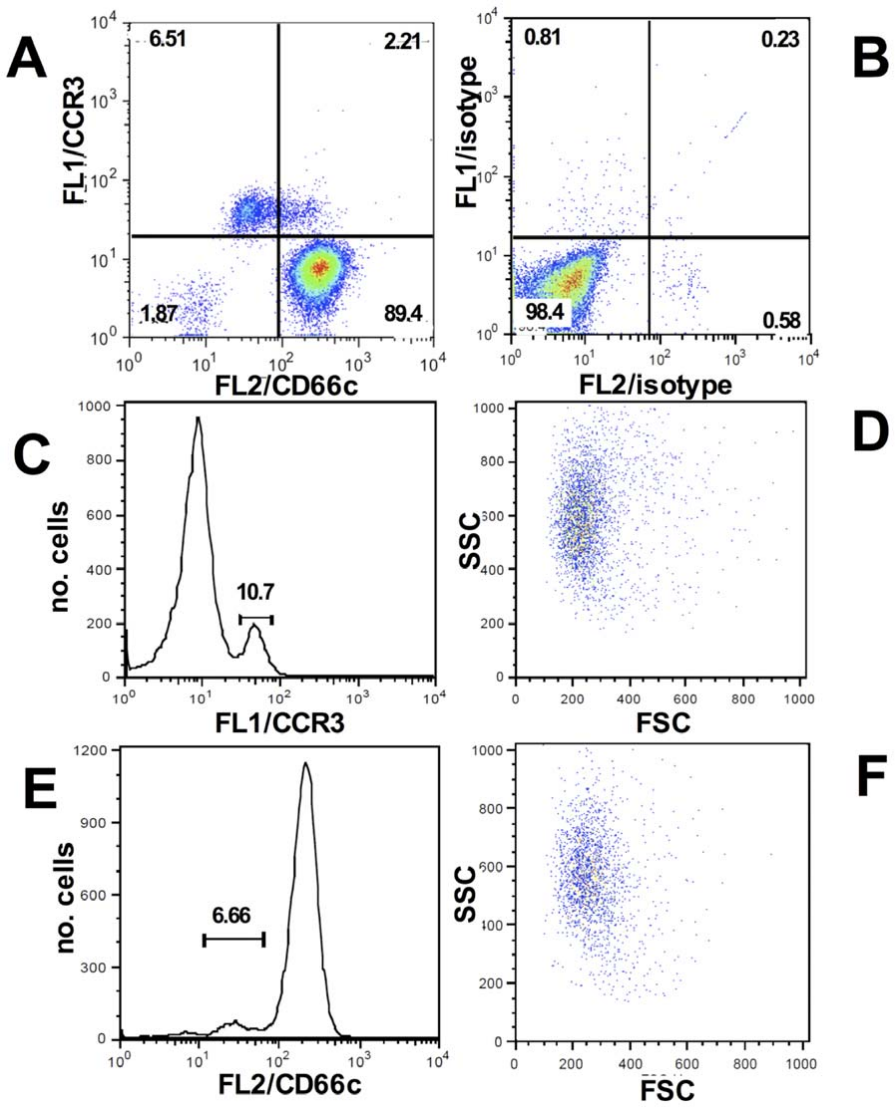
Identifying eosinophils in mixed granulocyte cultures. Cells were dual stained with CD66c-PE and CCR3-FITC and were analysed by flow cytometry. **A**, CD66c-PE (FL2-H axis) and CCR3-FITC (FL1-H axis) dual stained cells confirm a population of CCR3-positive/CD66c-low events (top left quadrant). **B**, Corresponding isotype controls. **C**, Identification of CCR3-positive events and the respective FSC/SSC profile of gated CCR3 positive events (**D**). **E**, Identification of CC66c-low events and the respective FSC/SSC profile of gated CC66c-low events (**F**).

Flow cytometry: Eosinophils were studied in mixed granulocyte populations using antibodies to CD66c-PE (BD Biosciences) as a positive marker for neutrophils which highly express CD66c compared to eosinophils which dimly stain for CD66c. This was verified by showing both that the CD66c-PE low events were also CCR3-FITC positive (when used), and that they fit a FSC-SSC profile consistent with that of eosinophils (fig. 1) [28]. Cells were washed in ice-cold PBS and stained with CD66c-PE and the vital dye ToPro®-3 iodide (Molecular Probes, Eugene, OR) as a measure of necrosis/cell lysis. Expression of phosphatidylserine (PS) on the outer leaflet of the plasma membrane is a well-established early marker of apoptosis [29]. Apoptotic eosinophils have been shown to bind to the phosphatidylserine probe Annexin V-FITC [30] and in selected experiments cells were also stained with Annexin V-FITC (BD Biosciences) to determine rates of apoptosis. Matched isotype control antibodies (mouse IgG1κ-PE and mouse IgG2a-FITC, BD Biosciences) were also used to confirm levels of non-specific binding. Countbright beads (Molecular Probes) were also acquired with each sample to measure total cell number. Data from 30,000 events were recorded from each sample by flow cytometry (FACScalibur, Becton Dickinson, CA) and analysed using FlowJo software (Tree Star Inc, Ashland, OR). Experiments where numbers of eosinophils fell below 500 events were not included.

Using this staining protocol it was possible to measure total eosinophil cell number and to assess the percentage of cells that were either apoptotic (Annexin V+ve/ToPro3-ve, dual positive) or necrotic (ToPro3+ve).

### Statistics

Statistical analysis was performed using PRISM v5 (GraphPad Software, San Diego, CA) and the tests indicated in the figure legend.

## Results

### S. aureus induces eosinophil cell death via a bacterial-associated factor

**Figure 2:**
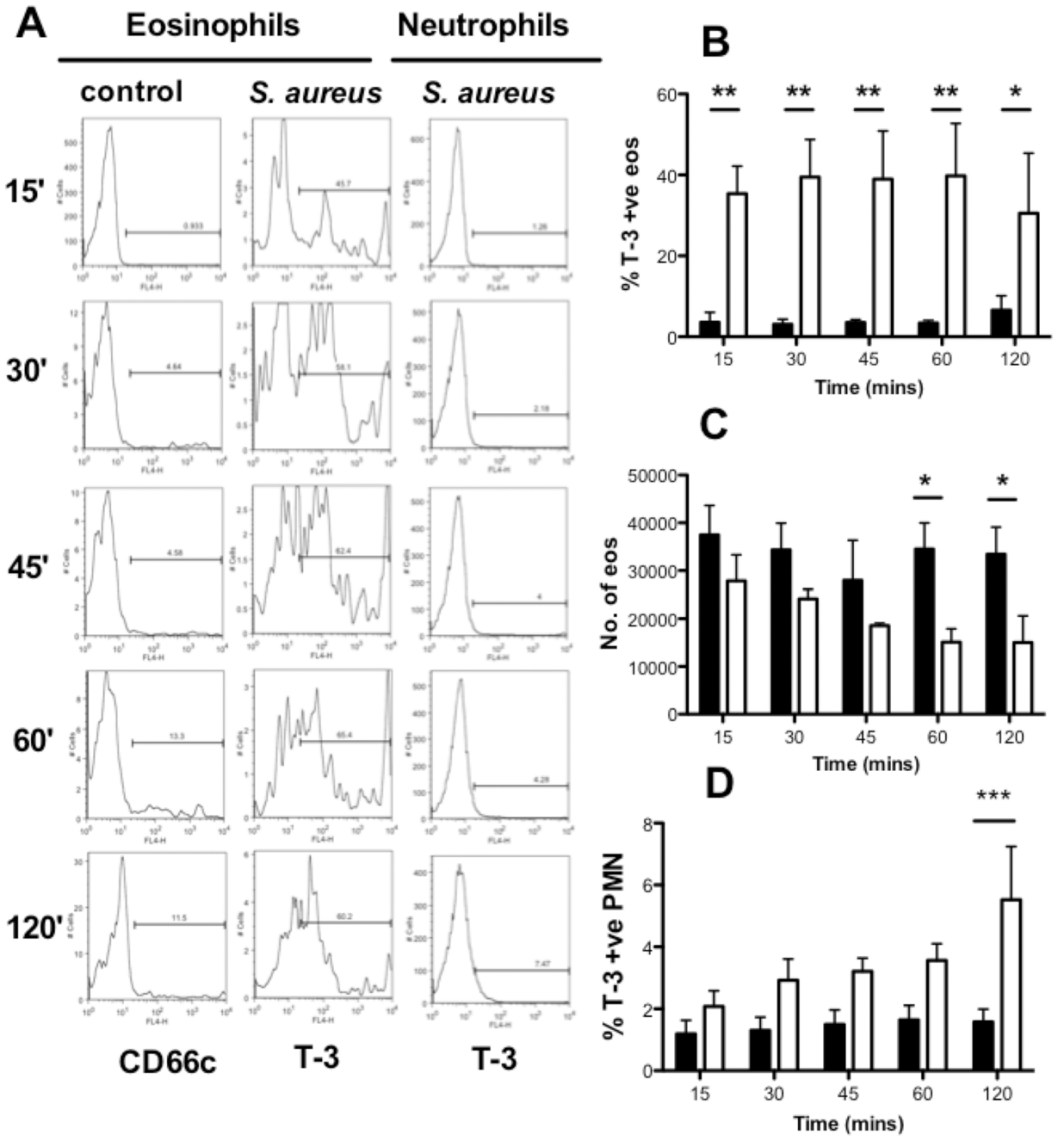
*S. aureus* supernatant rapidly induces eosinophil cell death. Mixed granulocytes were cultured with media (black bars) or *S. aureus* supernatant (10^−^3, open bars) for 15, 30, 45, 60 or 120 mins. Cells were stained with CD66c-PE and ToPro-3 and analysed by flow cytometry along with Countbright beads. Eosinophils were gated out as CD66c-PE low events (**A–C**). (**A**) representative flow cytometry histograms showing ToPro-3 staining of eosinophils and neutrophils following treatment with *S. aureus* supernatant. Charts show changes in percentage eosinophil ToPro-3 positivity (**B**), and total eosinophil number (**C**). (**D**) Shows percentage ToPro-3 positive neutrophils (CD66c high events). Data are shown as mean ±SEM from 3 independent experiments and statistically significant differences calculated by ANOVA with Bonferoni's post-test (*p≥0.05, **p≥0.01, *** p≥0.001).

The study of eosinophils in mixed granulocytes allowed the simultaneous study of two key inflammatory immune cells and their responses to an important human pathogen. Individual cell populations were identified within mixed granulocytes on the basis of their CD66c bright staining (neutrophils) or low staining (eosinophils, fig. 1). A fluorescent CCR3 antibody was not an appropriate method of staining eosinophils in these experiments since CCR3 signals were lost upon treatment with bacterial supernatant and therefore gating eosinophils as CCR3 positive events by flow was unreliable (data not shown). These studies revealed a significant difference between the granulocytes with respect to their sensitivity to *S. aureus*. Cells were treated with cell-free bacterial supernatant prepared from overnight growth of *S. aureus* in LB broth. Initial experiments to establish dilution ranges and time points revealed a potent and rapid pro-death effect of the supernatant on eosinophils and this was characterized using flow cytometry. Mixed granulocytes were cultured with media alone or with *S. aureus* supernatant (1 in 1,000 dilution) for time points up to 2 hours followed by staining with CD66c-PE and ToPro-3 and measurement of absolute cell loss using fluorescent beads. *S. aureus* supernatant induced significant eosinophil necrosis by 15′, with an increase in ToPro-3 positive events (fig. 2B) within the CD66c-low population. This was followed by significant loss of eosinophils at the later timepoint of 60 mins (3.5×10^4^±5.5×10^3^ eosinophils in control compared to 1.5×10^4^±2.8×10^3^ for supernatant treated cells, fig. 2C), suggesting cell necrosis precedes loss of cells from the population. The rapidity of eosinophils becoming ToPro3+ve suggested they were undergoing primary necrosis and this was confirmed by there being no detection of an Annexin V+ve/ToPro3-ve population preceding the appearance of necrotic cells (data not shown). In contrast, neutrophils were relatively unaffected by the bacterial supernatant, only showing a small yet significant increase in ToPro-3 positivity at 120 mins (fig. 2D). There was no detectable neutrophil cell loss at any timepoint (data not shown).

**Figure 3:**
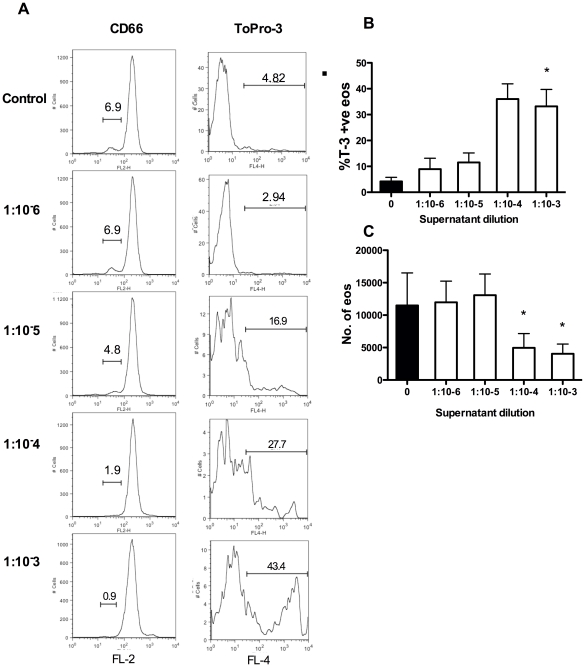
*S. aureus* supernatant induces eosinophil cell death at high dilutions. Mixed granulocytes were cultured with media (black bars) or a range of dilutions of *S. aureus* supernatant (open bars) for 2 hours. **A**, representative flow cytometry histograms showing loss of CD66c-low population and increases in ToPro-3 positivity of that population with supernatant treatment. Charts show changes in percentage eosinophil ToPro-3 positivity (**B**), and total eosinophil number (**C**). Data are shown as mean ±SEM from 7 independent experiments and statistically significant differences calculated by ANOVA with Dunnett's post-test (*p≥0.05).

**Figure 4:**
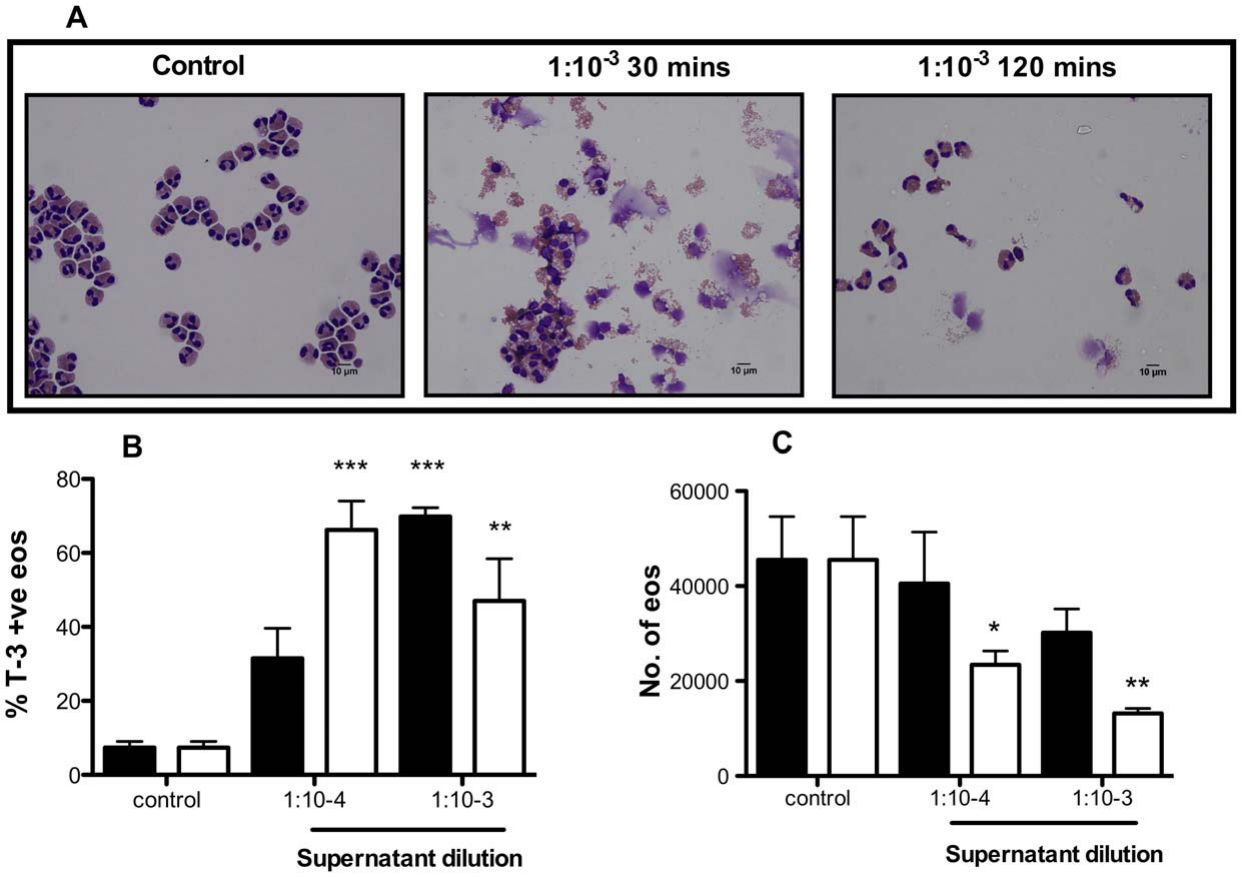
An equivalent degree of cell death is observed when eosinophils are treated in purified populations. Eosinophils were further purified from mixed leukocyte populations by negative magnetic selection yielding populations of 99±0.2% purity. Cells were incubated with media or *S. aureus* supernatant for 30 mins (black bars) or 120 mins (open bars) and ToPro-3 positivity and cell loss (Countbright beads) was measured by flow cytometry. **A**, representative light microscopy images of eosinophils undergoing cell death following treatment with media (control) or supernatant diluted at 1∶10^3^. Cells were sampled at 30 and 120 mins. Charts show changes in ToPro-3 positivity (**B**) and total cell number (**C**) from 3 independent experiments. Data are expressed at mean ± SEM and statistically significant differences between treatment and relevant control were calculated by ANOVA with Bonferoni post-test (*p≥0.05, **p≥0.01, *** p≥0.001).

Further experiments explored the lytic potency of the bacterial supernatants. Even at dilutions as high as 1 in 10,000, *S. aureus* supernatant significantly increased the percentage of ToPro-3 positive events (fig. 3B), which correlated with a significant reduction in the total number of eosinophils (fig. 3C). Eosinophils further purified from granulocyte populations by negative magnetic separation were also susceptible to an equivalent degree of cell death by *S. aureus* supernatant (fig. 4).

**Figure 5:**
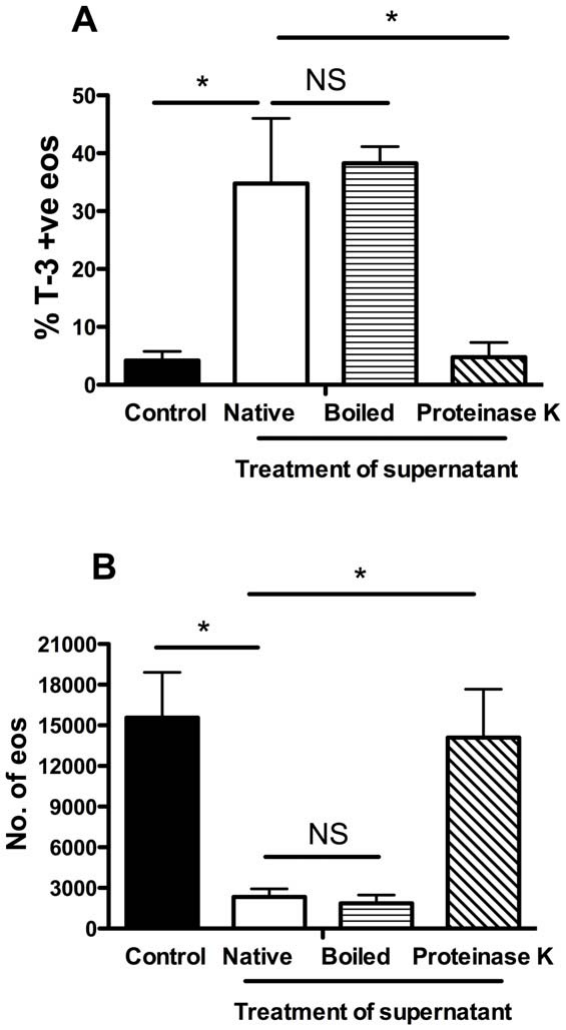
Pro-death effect of supernatant is lost with proteinase K treatment but is not affected by boiling. Mixed granulocytes were cultured with media (control, black bars), or *S. aureus* supernatant treated in the following way: untreated (native, open bars), boiled (horizontal lines) or proteinase K digestion (diagonal lines) for 2 hours. Cells were stained with CD66c-PE and ToPro-3 and analysed by flow cytometry along with Countbright beads. Eosinophils were gated out as CD66c-low events. Charts show changes in percentage eosinophil ToPro-3 positivity (**A**), and total eosinophil number (**B**). Data are shown as mean ±SEM from 3 independent experiments and statistically significant differences calculated by ANOVA with Bonferoni's post-test (*p≥0.05).

To determine whether the death-inducing factor(s) was of a proteinaceous nature, supernatant was heat-treated (100°C, 10 mins) or digested for 2 hours with proteinase K and the effect on eosinophil cell death examined (fig. 5). Although proteinase K treatment significantly abrogated the pro-death effect, heat-treated supernatant retained its ability to induce eosinophil cell death, suggesting the factor(s) is a heat-stable protein.

### Production of the pro-necrotic factor(s) is controlled by the agr locus

**Figure 6:**
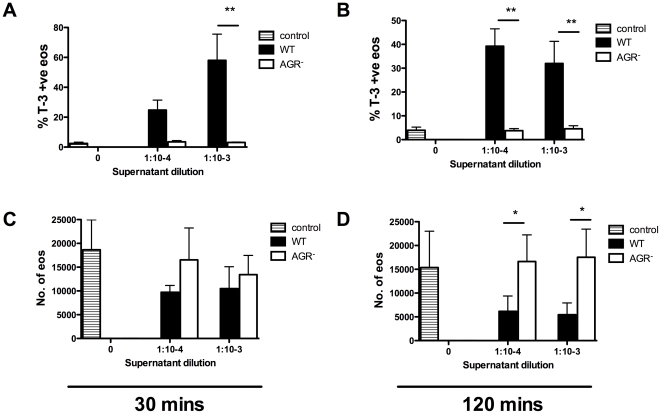
ToPro-3 positivity and cell loss is not seen in supernatants prepared from *S. aureus agr*. Mixed granulocytes were cultured with media (lined bars) or WT (closed bars) or *agr* (open bars) *S. aureus* supernatant at 1∶10^4^ and 1∶10^3^ for 30 (**A, C**) or 120 (**B, D**) mins. Cells were stained with CD66c-PE and ToPro-3 and were analysed alongside Countbright beads by flow cytometry. Eosinophils were gated out as CD66c-low events. Charts show changes in percentage eosinophil ToPro-3 positivity (**A, B**) and total eosinophil number (**C, D**). Data are shown as mean ±SEM from 4 independent experiments and statistically significant differences calculated by ANOVA with Bonferoni's post-test (*p≥0.05, **p≥0.01).

Since cellular effects similar to the ones we have observed are typically due to secreted bacterial toxins, we prepared supernatants from a *S. aureus* mutant deleted for the *agr* locus which controls the expression of key virulence factors [31]. These mutants lack expression of the α-, β-, δ-, and γ-hemolysins, leukocidins, enterotoxins and TSST-1 [31]. Bacterial cultures of SH1000 and its isogenic *agr* derivative were grown simultaneously and CFU counts shown to be comparable. Supernatant prepared from the *agr* deficient strain did not induce eosinophil ToPro-3 positivity (fig. 6A, B) or cell loss (fig. 6C, D) in contrast to the WT supernatant, suggesting production of the pro-death factor(s) requires the *agr* locus.

### An Hla deficient supernatant fails to induce eosinophil cell death

**Figure 7:**
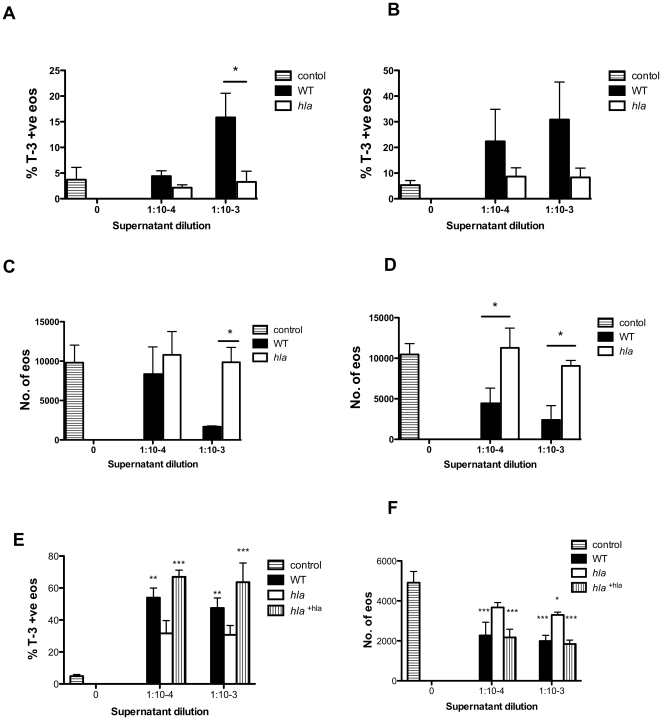
Supernatant prepared from a Hla mutant (*hla*) does not induce eosinophil cell death. Mixed granulocytes were cultured with media (lined bars) or a range of dilutions of WT (closed bars) or *hla* (open bars) *S. aureus* supernatant for 30 (**A, C**) or 120 (**B, D**) mins. Cells were stained with CD66c-PE and ToPro-3 and were analysed alongside Countbright beads by flow cytometry. Eosinophils were gated out as CD66c-low events. Charts show changes in percentage eosinophil ToPro-3 positivity (**A, B**) and total eosinophil number (**C, D**). In separate experiments mixed granulocytes were also incubated with supernatants from plasmid HLA complemented *hla* (*hla* ^+hla^ vertical lined bars) for 120 mins. Cells were stained with CD66c-PE and ToPro-3 and were analysed as described above. Charts show changes in percentage eosinophil ToPro-3 positivity (**E**) and total eosinophil number (**F**). Data are shown as mean ±SEM from 3 independent experiments and statistically significant differences calculated by ANOVA with Bonferoni's post-test (*p≥0.05, **p≤0.01, ***p≤0.001).

**Figure 8:**
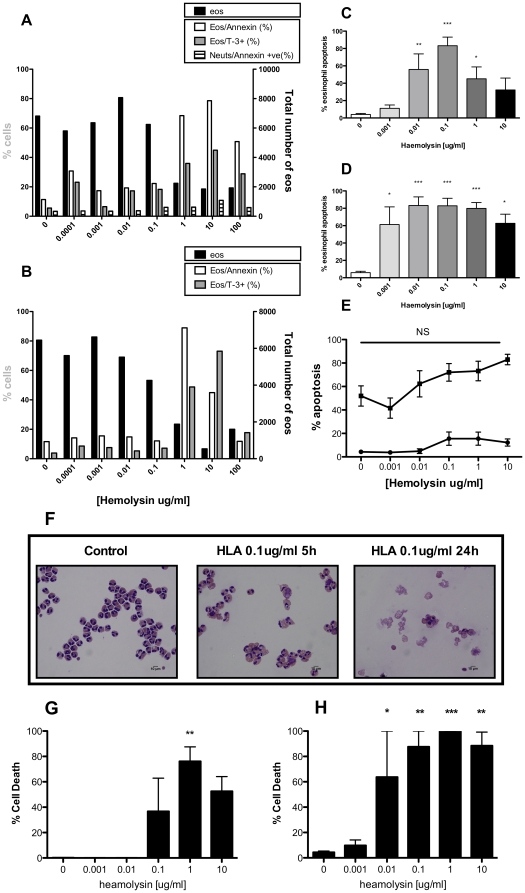
Purified Hla induces eosinophil apoptosis. Mixed granulocytes were cultured with a concentration response of purified *S. aureus* Hla and cell death assessed. **A, B**, In a single expt, cells were harvested after 1 (**A**) and 3 hours (**B**), stained with CD66c-PE, Annexin-FITC and ToPro-3 and analysed alongside Countbright beads by flow cytometry. Eosinophils were gated out as CD66c-low events. Charts show changes in total number of eosinophils (closed bar, right axis), percentage eosinophil Annexin positivity (open bars, left axis), neutrophil Annexin positivity (striped bars, left axis **A** only) and percentage eosinophil ToPro-3 positivity (shaded bars, left axis). **C, D,** In separate experiments, cells were harvested after 5 (**C**) or 24 hours (**D**) and eosinophils assessed by light microscopy for morphological changes of apoptosis. Neutrophil apoptosis was also assessed in these experiments (**E**). In separate experiments, eosinophils further purified by negative magnetic selection were also incubated with Hla for 5 (**G**) or 24 (**H**) hours and cells observed by light microscopy. Representative images shown in **F**. Data are shown as mean ±SEM from 4 (**C–E**) or 3 (**G**–**H**) independent experiments and statistically significant differences calculated by ANOVA with Dunnett's post-test (*p≥0.05, **p≥0.01, ** p≥0.001).

We hypothesised that Hla played a key role in this rapid necrosis since this toxin is a key cytolytic factor, is controlled by the *agr* locus and Hla-positive strains are commonly isolated from patients with eosinophillic diseases such as atopic dermatitis [32], [33]. To test this, supernatant was prepared from an Hla deficient strain (*hla*) and eosinophil cell death assessed after 30 and 120 mins. Figure 6 shows that, despite profound effects of the WT supernatant, the *hla* supernatant did not cause ToPro-3 positivity (fig. 7A, B) or cell loss (fig. 7C, D) compared to the WT counterpart at either time point. It was observed that more concentrated preparations (1 in 100 dilution) of *hla* supernatant induced moderate eosinophil cell death in these assays (3.7±2.3% ToPro-3 positivity in control vs 12.6±5.9% in *hla* supernatant diluted 1 in 100 after 30 mins) that was comparable to the profound cell death seen with substantially more diluted WT supernatants (data not shown). In genetic complementation experiments, the *hla* mutant was genetically reconstituted with a plasmid expressing Hla to further demonstrate the eosinophil killing abilities of Hla in bacterial supernatant. Figure 7E, F show treatment with *hla* ^+hla^ supernatant results in a significant increase in ToPro-3 positivity and significant decrease in eosinophil number respectively, therefore restoring the death-inducing capacity of the Hla deficient supernatant. To provide further confirmation that Hla was a potent eosinophil lysogen, granulocytes were incubated with a concentration range of purified Hla for 1 (fig. 8A) and 3 hours (fig. 8B) and eosinophil cell death assessed by flow cytometry. Eosinophils, gated as CD66c-low events, stained positively with Annexin-V and ToPro-3 at concentrations of Hla ≥1 µg/ml (fig. 8A, B: open bars and shaded bars respectively). This staining was accompanied by a drop in total cell number indicating these apoptotic/secondary necrotic cells were undergoing lysis (fig. 8A, B: closed bars). Since dual positivity of Annexin-V and ToPro-3 can indicate that cells have either undergone apoptosis or necrosis (when Annexin-V stains the PS on the inner membrane), we examined Hla treated eosinophils by light microscopy for morphological changes associated with apoptosis after 5 (fig. 8C) and 24 hours (fig. 8D). Eosinophils displayed distinct morphological changes of apoptosis, including nuclear condensation and cell shrinkage and this reached significance at 100 ng/ml Hla at 5 hours (fig. 8C) and 1 ng/ml at 24 hours (fig. 8D). The bell-shaped nature of the concentration response curve further supports the hypothesis that cells are lost at higher concentrations (≥1 µg/ml) of Hla. In contrast, neutrophil apoptosis as assessed by Annexin-V positivity (fig. 7A) and morphology (fig. 8E), was modestly increased and not statistically significant at either timepoint. The pro-death effect of Hla was confirmed in purified populations of eosinophils (fig. 8F, G, H), where statistical significance was achieved at 1 µg/ml Hla at 5 hours and 10 ng/ml at 24 hours, suggesting that although still profoundly cytotoxic, Hla was less potent when treating pure populations of eosinophils.

## Discussion

Bacterial colonization with *S. aureus* is a prominent feature of conditions such as atopic dermatitis [34], [35], with a correlation between numbers of bacteria present on the skin and disease severity. Furthermore, anti-microbial treatments lead to improvement in skin lesions, suggesting a causal relationship [36], [37]. *S. aureus* has also been implicated in the development of nasal polyps [19], [20] and a role for staphylococcal enterotoxins has been described in asthma [38].

In these studies we describe the ability of *S. aureus* to profoundly reduce eosinophil viability via an Hla dependent mechanism. Pro-death activity was observed in heat-treated supernatant, indicating it was attributable to a heat-stable factor(s) and in support of this, Hla has been shown to have a high tolerance to heat [39]. This together with the loss of activity after proteinase K treatment confirmed that the pro-death factor was a heat-resistant protein.

Hla is a clinically important pore forming toxin which is produced by the vast majority of clinical strains of *S. aureus* [40]. It has been implicated in many diseases, in particular pneumonia, and septic arthritis [41], [42], [43], as well as brain abscesses [44]. Secreted as a 33 kDa monomer, Hla binds to the target cell plasma membrane and oligomerises into hexamers or heptamers before inserting into the lipid bilayers to form pores [45]. Cell toxicity is rapidly achieved due to the destruction of the plasma membrane, leak of cellular ions or via delivery of toxic compounds through the pores. The concentration of Hla is critical for governing the mode of death: at low doses (Hla<100 ng/ml) cell death is typically via caspase-mediated apoptosis whereas at higher concentrations cell death occurs by necrotic mechanisms [46], [47], [48], [49], [50], [51]. The effects of Hla are also time dependent; in epithelial cells a transient phase of apoptosis is followed by cellular necrosis [52]. On the whole, Hla is thought to predominantly induce necrosis, particularly when present with other bacterial factors. In our studies we have shown that in isolation, Hla induces eosinophil apoptosis (determined by morphological changes and PS exposure measured by Annexin-V positivity) at low concentrations (<100 ng/ml) and early time points (<5 hours), but induces necrosis and subsequent cell loss at concentrations in excess of 1 µg/ml. It is worth noting that standard Hla preparations contain some impurities and we cannot exclude the possibility that other agents in the Hla may have contributed to the cytotoxicity observed. When present in bacterial supernatant, Hla appears to contribute to a principally necrotic death, characterised by rapid ToPro-3 positivity occurring within 15 mins and ensuing cell loss by 2 hours. This is likely to be the result of high concentrations of Hla that have accumulated during the overnight growth period. It could also be attributable to other cellular disrupting factors that may act either to sensitise the cell to pore formation by damaging the plasma membrane or to mediate cellular damage by a different mechanism. This is supported by the ability of the Hla deficient mutant to cause moderate levels of eosinophil cell death with more concentrated supernatant preparations, suggesting a minor role for other factors and a dominant role for Hla. It is worth noting that other factors produced by other *S. aureus* strains with alternative toxin profiles may also have a role in eosinophil-induced cell toxicity however, based on the findings in this study, we show that the critical factor in *S. aureus*-induced eosinophil cell death is Hla.

Interestingly, we showed neither *S. aureus* supernatants nor purified Hla induced death of neutrophils granulocytes. In support of this, published data confirm neutrophils are remarkably resistant to cell death mediated by Hla [53], with Hla unable to form a pore in neutrophil cell membranes. The different susceptibility of these two closely-related cell types suggests neutrophil resistance is likely to represent a host defence mechanism evolved to provide effective defence against bacteria producing lytic toxins such as Hla. *S. aureus* supernatant was able to induce modest but statistically significant levels of neutrophil ToPro-3 positivity at late time points. This could be due to the fact that neutrophils are relatively, rather than absolutely resistant to Hla, or attributable to the effects of high levels of eosinophil lysis within the mixed granulocyte populations (causing damage to the neutrophil plasma membrane and therefore uptake of the vital dye) rather than being a direct effect of Hla. It is yet to be elucidated whether the eosinophil cell death is a host- or pathogen- mediated process. The rapid and pro-necrotic death suggests a pathogen-mediated phenomenon, both since death by apoptosis would be a preferable mechanism for the host and since *in vivo* disease models have shown Hla mutants have reduced virulence [43], [54], [55], [56], [57]. Recent animal models clearly demonstrate that Hla contributes to disease pathogenesis in *S. aureus* skin infections, where Hla deficient strains result in smaller skin lesions compared to wild type strains and that vaccination against Hla reduces disease severity [56], [57]. In addition, the dermonecrosis visible following infection by wild type strains is not seen in Hla negative mutants, which supports a pro-necrotic role for Hla *in vivo* [56]. Nonetheless, host benefits are also possible: eosinophil cationic protein (ECP) has been shown to have bactericidal effects against *S. aureus* [14] so that eosinophil lysis at the site of infection could contribute to infection control. A more likely scenario *in vivo* is that high local concentrations of Hla at the site of infection, in combination with other potentially cytolytic factors, would lead to the necrotic death of tissue eosinophils, with consequent release of injurious granule contents causing damage to the surrounding cells and perpetuating inflammation [58]. This unfavourable host situation may be of some benefit to the pathogen in that local immune responses may be hindered by the necrotic debris and loss of tissue architecture would favour further colonisation and allow dissemination of infection. We also speculate that lysis of tissue eosinophils by Hla would lead to the liberation of pre-formed pools of the eosinophil chemoattractants IL-6 and RANTES, therefore recruiting further eosinophils to the area [59]. These phenomena could potentially explain the tissue eosinophilia observed in *S. aureus* disease.

These data are the first example of a pro-necrotic effect of Hla on human eosinophils, and may in part explain tissue inflammation seen in *S. aureus* colonisation of allergic disease.
